# Cystatin C and sarcopenia index are associated with cardiovascular and all-cause death among adults in the United States

**DOI:** 10.1186/s12889-024-19137-x

**Published:** 2024-07-23

**Authors:** Tianbo Wang, Yuxin Zhu, Xiaohan Liu, Yue Zhang, Zhen Zhang, Jing Wu, Gang Huang, Junbo Xu

**Affiliations:** 1 The Third People’s Hospital of Chengdu, Affiliated Hospital of Southwest Jiaotong University, College of Medicine, Southwest Jiaotong University, Chengdu, 610031 Sichuan China; 2 Department of Cardiology, The Third People’s Hospital of Chengdu, Chengdu, 610014 Sichuan China; 3 Department of Geriatric, The Third People’s Hospital of Chengdu, Chengdu, 610014 Sichuan China

**Keywords:** Cystatin C, Creatinine, Creatinine to cystatin C ratio, Sarcopenia index, Cardiovascular death

## Abstract

**Objectives:**

This study aimed to investigate the association of cystatin C, serum creatinine and sarcopenia index with cardiovascular and all-cause death in general population.

**Methods:**

Data of participants from the National Health and Nutrition Examination Surveys (NHANES) from 1999 to 2004 were used and all participants were followed up regularly until December 31, 2019. Multivariable Cox analysis was used to investigate the association of cystatin C, serum creatinine and sarcopenia index with cardiovascular and all-cause death. Restricted cubic spline was conducted to evaluate the nonlinear association.

**Results:**

A total of 9894 participants with a mean age of 45.64 years were enrolled and followed up for a mean duration of 15.62 ± 4.68 years. Approximately 50.3% were male and there were a total of 2681 all-cause deaths and 691 cardiovascular deaths recorded during the follow-up period. In final adjusted model, compared with the first quartile of cystatin C (< 0.659 mg/L), the risk of cardiovascular and all-cause death increased 2.36-fold and 1.71-fold for participants in the fourth quartile (≥ 0.877 mg/L) (HR: 3.36, 95% CI: 2.06–5.46, *P* < 0.001; HR: 2.71, 95% CI: 2.17–3.38, *P* < 0.001; respectively). Furthermore, a higher sarcopenia index (< 88.41 vs. ≥125.52) was associated with the reduced risk of cardiovascular death (HR: 0.41, 95% CI: 0.31–0.53, *P* < 0.001) as well as all-cause death (HR: 0.41, 95% CI: 0.35–0.49, *P* < 0.001). Additionally, restricted cubic splines showed that there was a nonlinear relationship between sarcopenia index levels and all-cause death while there was a linear relationship between sarcopenia index levels and cardiovascular death.

**Conclusions:**

Higher sarcopenia index was associated with the decreased risk of cardiovascular and all-cause death in general population in the United States. Elevated cystatin C was positively associated with cardiovascular and all-cause death.

**Supplementary Information:**

The online version contains supplementary material available at 10.1186/s12889-024-19137-x.

## Introduction

Cardiovascular disease (CVD) is recognized as prominent contributors to increasing global mortality and disability, as substantiated by an escalating mortality rate over time [1, 2]. It is estimated that the overall prevalence of CVD witnessed a substantial increase from 271 million in 1990 to 523 million in 2019, meanwhile CVD-related fatalities also increased from 12.1 million in 1990 to 18.6 million in 2019 [3]. CVD is frequently associated with comorbidities such as diabetes, dyslipidemia, hypertension, obesity, cigarette smoking, physical inactivity, and malnutrition. Hence, the identification and prompt management of modifiable risk factors hold paramount significance.

Cystatin C, a cysteine protease inhibitor presents in various bodily fluids, including plasma, is ubiquitously distributed. Cystatin C is also consistently produced in nucleated cells, freely filtered by the glomerulus, unaffected by muscle mass, and entirely reabsorbed and metabolized by the proximal renal tubules [4, 5]. Consequently, cystatin C exhibits less susceptibility to age, sex, race, diet, nutritional status and so on in comparison to creatinine. Creatinine is commonly used as a surrogate indicator for muscle mass due to its association with creatine in muscles [6]. The serum creatinine to cystatin C ratio (Cre/CysC) has emerged as a potential alternative marker for assessing muscle mass in the body. Sarcopenia, a generalized and progressive muscle disease, is associated with an increased risk of adverse outcomes such as falls, fractures, CVD, and mortality [7]. Therefore, a named sarcopenia index (SI) was developed and used to estimate muscle mass, which is defined by the serum Cre/CysC ratio x 100 [8, 9].

Studies have documented the correlation between cystatin C and serum creatinine and death among the elderly [10]. Furthermore, cystatin C has been demonstrated to have a superior predictive ability for death in patients with CVD [11–13]. Additionally, studies have investigated the association between SI and all-cause death in various population, including elderly individuals and individuals with coronary heart disease, heart failure and cancer [14–18]. However, it is worth noting that these studies are limited to specific populations or solely focused on the correlation between SI and all-cause death. Limited research has focus on the relationship between cystatin C, serum creatinine, and SI with long-term health outcomes, particularly in relation to cardiovascular health. Shi et al. has discovered an association between all-cause death and Cre/CysC using NHANES 1999–2002 data but have not observed an association with cardiovascular death [9]. Hence, this study primarily aimed to investigate the potential relationship between cystatin C, serum creatinine, and SI and cardiovascular and all cause death in general population.

## Methods

### Study population

**Fig. 1:**
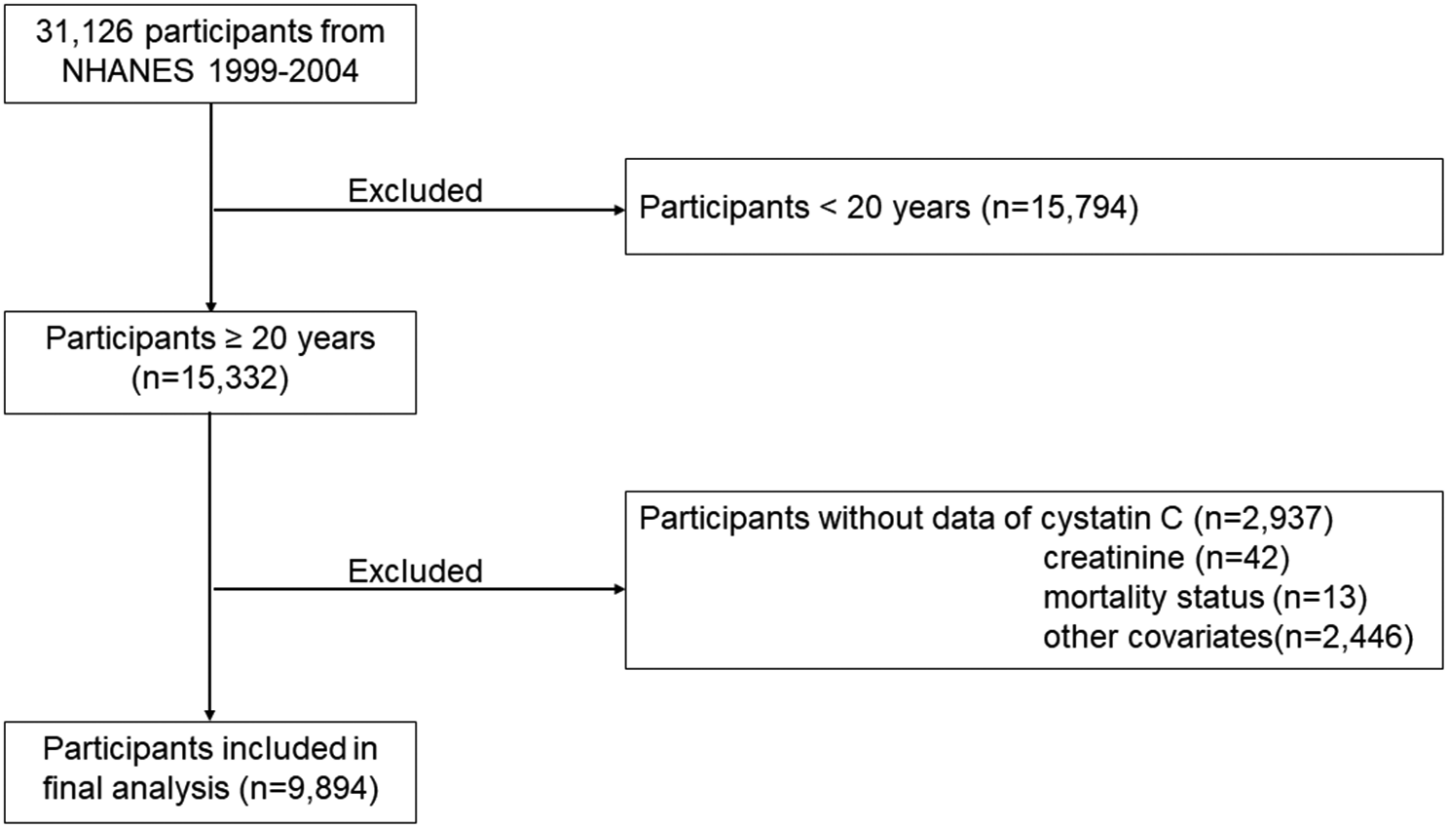
Flow chart of this study

This study population and their data comes from three cycles of the National Health and Nutrition Examination Survey (NHANES) between 1999 and 2004 (https://www.cdc.gov/nchs/nhanes/index.htm). NHANES is a comprehensive and ongoing survey that aimed to investigate the health and nutritional status of adults and children in the United States, encompassing a nationally representative sample of non-institutionalized US citizen. To ensure the integrity of this analysis, participants younger than twenty years old (*n* = 15,794), with missing data of cystatin C (*n* = 2937), serum creatinine (*n* = 42), mortality status (*n* = 13), and other important covariates (*n* = 2446) were excluded. Finally, 9894 participants were enrolled in the study (Fig. 1).

### Cystatin C and serum creatinine

Cystatin C was tested from stored surplus specimens in the NHANES 1999–2004 cycles and measured in serum using a cystatin C immunoassay (Siemens Healthcare Diagnostics) on an automated multichannel analyser, Siemens Dimension Vista 1500 (Siemens Healthcare Diagnostics). The measurement of serum creatinine was based on the work of Popper et al. and Seeling and Wuest utilizing the Jaffé reaction.

### Cardiovascular and all cause death

The outcomes variables of this study were defined as cardiovascular and all-cause death. Participants were followed up regularly until the end of December 2019 and data about cardiovascular and all cause death were extracted from the National Death Index (https://www.cdc.gov/nchs/data-linkage/mortality-public.htm). All-cause death was defined according to the International Classification of Diseases, 10th revision (ICD-10), as death resulting from any cause. Cardiovascular death was defined as death resulting from any CVD according to ICD-10 codes I00-I09, I11, I13, or I20-I51.

### Covariates

Variables included sex, age, race (Mexican American, Other Hispanic, Non-Hispanic White, Non-Hispanic Black, Other Races), educational level (< high school, high school, and > high school), smoking status (current, former, and never), drinking status (current, former, and never), physical activity, body mass index, hypertension, diabetes mellites and CVD. Other continuous variables included alanine aminotransferase (U/L), albumin (g/L), total cholesterol (mmol/L), high density lipoprotein (mmol/L), blood urea nitrogen (mmol/L), blood uric acid (µmol/L) and serum glucose (mmol/L). Participants with hypertension for any of the following reasons: systolic blood pressure ≥ 140 mmHg and/or diastolic blood pressure ≥ 90 mmHg, or any self-reported taking anti-hypertensive medications now. Diabetes mellites was defined as a history of diabetes, or any self-reported taking insulin, or other diabetes medications now, or a fasting glucose level ≥ 7mmol/L (126 mg/dL). CVD was defined as the participants were asked, “Has a doctor or other health professional ever told you that you have congestive heart failure /coronary heart disease /angina pectoris/heart attack/stroke?”. Drinking status was defined through the questionnaire, “Had at least 12 alcohol drinks/lifetime?” and “How often drink alcohol over past 12 months”. Smoking status was defined through the questionnaire, “Smoked at least 100 cigarettes in life” and “Do you now smoke cigarettes”. Physical activity was defined through the questionnaire, “Tasks around home/yard past 30 days” [19].

### Statistical analysis

Weighted data are included in final statistical analysis. Data are shown as mean ± standard deviation (SD) for continuous variables and number (n) with percentage (%) for categorical variables. The chi-square test and Kruskal‒Wallis test were used to compare the difference of frequencies and continuous variables between groups, respectively. Multivariable Cox regression Models were performed to investigate the association of cystatin C, serum creatinine and sarcopenia index with cardiovascular and all-cause death. The Kaplan–Meier analysis was performed to estimate the cumulative event incidence of cardiovascular and all-cause death between groups. Age and sex were adjusted in Model (1) Age, sex, race/ethnicity, education, smoking status, alcohol consumption, physical activity, and body mass index were adjusted in Model (2) Age, sex, race/ethnicity, education, smoking status, drinking status, physical activity, body mass index, total cholesterol, high density lipoprotein, albumin, alanine aminotransferase, blood urea nitrogen, blood uric acid, glucose, hypertension, diabetes mellites, and CVD were adjusted in Model (3) Restricted cubic splines with Model 3 were employed to evaluate the nonlinear association of cystatin C, serum creatinine and sarcopenia index with cardiovascular and all-cause death. Subgroup analysis with Model 3 based on sex, age, race, body mass index, hypertension, diabetes mellites, and CVD were conducted. Additionally, Cystatin C (< 0.659, < 0.753, < 0.877 and ≥ 0.877), serum creatinine (< 0.7, < 0.8, < 1.0 and ≥ 1.0) and SI (< 88.41, < 106.24, < 125.52 and ≥ 125.52) were divided into quartiles for analysis. All statistical analysis were performed using R version 4.2.3, and a two tailed *p* < 0.05 was considered statistically significant.

## Result

### Population characteristics

Totally, 9894 participants were included in the final analysis and followed up for a mean duration of 15.62 ± 4.68 years. A total of 2681 participants died, in which 691 died from cardiovascular events. There were no obvious differences between participants with missing covariates and those with excluded important covariates (Table 1). The mean age of all participants was 45.64 ± 16.59 years and 50.3% of participants were male. The mean levels of cystatin C, serum creatinine and SI were 0.79 ± 0.29 mg/L, 0.85 ± 0.37 mg/dL, and 111.07 ± 31.31, respectively (Table 1). Participants with the highest cystatin C levels were older and more likely to be male, non-Hispanic White and obese individuals than those with lower cystatin C levels (Table 2). In addition, participants with elevated levels of cystatin C had a higher total cholesterol, higher blood urea nitrogen, higher blood uric acid, higher serum glucose and lower levels of high density lipoprotein.

**Table 1:** Baseline characteristics of the study population with/without miss covariates

	All participants with missing covariates (*n* = 12, 340)	Participants included in final analysis (*n* = 9894)
Number of participants	199,132,062	163,477,895
Age, years	46.14 ± 16.91	45.64 ± 16.59
Male	95,198,642 (47.8%)	82,178,874 (50.3%)
Age		
< 40 years	78,780,154 (39.6%)	65,856,078 (40.3%)
40–59 years	75,474,561 (37.9%)	62,354,663 (38.1%)
≥ 60 years	44,877,347 (22.5%)	35,267,154 (21.6%)
Race/ethnicity		
Mexican American	14,517,777 (7.3%)	11,673,308 (7.1%)
Other Hispanic	11,410,249 (5.7%)	8,836,080 (5.4%)
Non-Hispanic White	142,548,296 (71.6%)	119,728,004 (73.2%)
Non-Hispanic Black	21,666,998 (10.9%)	16,518,737 (10.1%)
Other race	8,988,743 (4.5%)	6,721,767 (4.1%)
Education		
< High school	40,765,236 (20.5%)	30,851,828 (18.9%)
High school	52,054,228 (26.2%)	42,613,853 (26.1%)
> High school	105,993,370 (53.3%)	90,012,215 (55.1%)
Smoking		
Never	99,571,847 (50.1%)	80,350,034 (49.2%)
Former	50,387,707 (25.3%)	42,138,099 (25.8%)
Current	48,922,841 (24.6%)	40,989,762 (25.1%)
Drinking		
Never	23,977,828 (13.8%)	21,637,240 (13.2%)
Former	15,375,397 (8.8%)	14,001,542 (8.6%)
Current	134,990,466 (77.4%)	127,839,112 (78.2%)
Physical activity	126,589,428 (64.9%)	108,305,451 (66.3%)
Body mass index		
< 30	134,242,701 (68.9%)	114,225,835 (69.9%)
≥ 30	60,509,824 (31.1%)	49,252,060 (30.1%)
Hypertension	58,542,172 (29.8%)	47,458,008 (29.0%)
Diabetes mellites	17,036,428 (8.6%)	13,040,417 (8.0%)
Cardiovascular disease	1,7438,648 (8.8%)	12,721,858 (7.8%)
TC, mmol/L	5.24 ± 1.09	5.24 ± 1.09
HDL, mmol/L	1.35 ± 0.41	1.35 ± 0.41
ALT, U/L	26.02 ± 30.35	26.51 ± 32.71
Albumin, g/L	43.27 ± 3.45	43.43 ± 3.42
BUA, µmol/L	318.76 ± 86.21	319.90 ± 85.25
BUN, mmol/L	4.81 ± 1.92	4.79 ± 1.84
Cystatin C, mg/L	0.79 ± 0.30	0.79 ± 0.29
Serum creatinine, mg/dL	0.85 ± 0.39	0.85 ± 0.37
Sarcopenia index	109.95 ± 31.31	111.07 ± 31.31

Data are presented as means ± SD or n (%)

TC, Total Cholesterol; HDL, High Density Lipoprotein; ALT, Alanine Aminotransferase; BUN, Blood Urea Nitrogen; BUA, Blood Uric Acid

**Table 2:** Baseline characteristics of the study population according to quartile of Cystatin C

	Cystatin C (mg/L)				
	Q1 < 0.659	Q2 < 0.753	Q3 < 0.787	Q4 ≥ 0.787	*P* value
Number of participants	2465	2466	2480	2483	
Age, years	37.69 ± 11.67	41.41 ± 13.49	46.39 ± 15.94	59.88 ± 17.11	< 0.001
Male	826 (32.6%)	1311 (51.9%)	1478 (61.4%)	1388 (54.3%)	< 0.001
Age					< 0.001
< 40 years	1492 (59.0%)	1,083 (46.9%)	747 (36.3%)	232 (14.2%)	
40–59 years	769 (36.8%)	920 (42.8%)	820 (41.1%)	447 (29.5%)	
≥ 60 years	204 (4.2%)	463 (10.3%)	913 (22.6%)	1804 (56.2%)	
Race/ethnicity					< 0.001
Mexican American	739 (12.3%)	621 (8.3%)	475 (4.7%)	375 (2.7%)	
Other Hispanic	129 (6.6%)	101 (5.1%)	125 (6.0%)	82 (3.7%)	
Non-Hispanic White	938 (59.9%)	1216 (72.1%)	1451 (79.0%)	1634 (83.0%)	
Non-Hispanic Black	553 (15.3%)	444 (10.2%)	362 (7.4%)	329 (7.3%)	
Other race	106 (5.9%)	84 (4.3%)	67 (2.9%)	63 (3.3%)	
Education					< 0.001
< High school	705 (17.6%)	683 (15.9%)	725 (17.8%)	909 (25.8%)	
High school	509 (21.1%)	584 (25.4%)	625 (27.8%)	633 (30.5%)	
> High school	1,251 (61.3%)	1,199 (58.6%)	1,130 (54.4%)	941 (43.7%)	
Smoking					< 0.001
Never	1,506 (59.9%)	1,271 (51.2%)	1,103 (43.1%)	1,057 (41.5%)	
Former	498 (20.9%)	614 (24.9%)	704 (25.9%)	912 (32.5%)	
Current	461 (19.1%)	581 (23.9%)	673 (31.0%)	514 (26.0%)	
Drinking					< 0.001
Never	438 (14.6%)	313 (11.0%)	318 (10.5%)	482 (18.3%)	
Former	220 (6.9%)	198 (7.0%)	225 (7.7%)	353 (13.7%)	
Current	1,807 (78.5%)	1,955 (82.0%)	1,937 (81.7%)	1,648 (68.0%)	
Physical activity	1,462 (66.5%)	1,475 (68.2%)	1,544 (69.0%)	1,332 (59.7%)	< 0.001
Body mass index					< 0.001
< 30	1,873 (80.0%)	1,731 (71.6%)	1,612 (65.4%)	1,572 (61.3%)	
≥ 30	592 (20.0%)	735 (28.4%)	868 (34.6%)	911 (38.7%)	
Hypertension	391 (13.8%)	634 (21.7%)	926 (30.3%)	1,553 (55.5%)	< 0.001
Diabetes mellites	186 (5.0%)	189 (5.4%)	275 (7.2%)	488 (15.9%)	< 0.001
Cardiovascular disease	56 (1.9%)	116 (3.6%)	223 (7.1%)	618 (21.4%)	< 0.001
TC, mmol/L	5.10 ± 1.04	5.19 ± 1.10	5.31 ± 1.06	5.40 ± 1.14	< 0.001
HDL, mmol/L	1.50 ± 0.42	1.35 ± 0.40	1.29 ± 0.38	1.26 ± 0.39	< 0.001
ALT, U/L	23.40 ± 14.36	27.43 ± 29.95	28.13 ± 20.27	26.83 ± 56.55	< 0.001
Albumin, g/L	43.46 ± 3.51	43.77 ± 3.27	43.64 ± 3.35	42.67 ± 3.49	< 0.001
BUA, µmol/L	276.39 ± 74.77	311.59 ± 78.34	335.70 ± 79.79	362.16 ± 86.76	< 0.001
BUN, mmol/L	4.10 ± 1.38	4.52 ± 1.36	4.80 ± 1.41	5.96 ± 2.65	< 0.001
Glucose, mmol/L	5.09 ± 1.57	5.16 ± 1.47	5.18 ± 1.27	5.58 ± 1.89	< 0.001
Cystatin C, mg/L	0.59 ± 0.06	0.70 ± 0.03	0.81 ± 0.04	1.10 ± 0.49	< 0.001
Serum creatinine, mg/dL	0.73 ± 0.17	0.81 ± 0.18	0.87 ± 0.19	1.05 ± 0.69	< 0.001
Sarcopenia index	124.47 ± 42.51	115.13 ± 25.52	107.41 ± 23.11	94.43 ± 22.30	< 0.001

Data are presented as means ± SD or n (%)

TC, Total Cholesterol; HDL, High Density Lipoprotein; ALT, Alanine Aminotransferase; BUN, Blood Urea Nitrogen; BUA, Blood Uric Acid

### Risk of cardiovascular and all-cause death

#### Cystatin C and serum creatinine

**Fig. 2:**
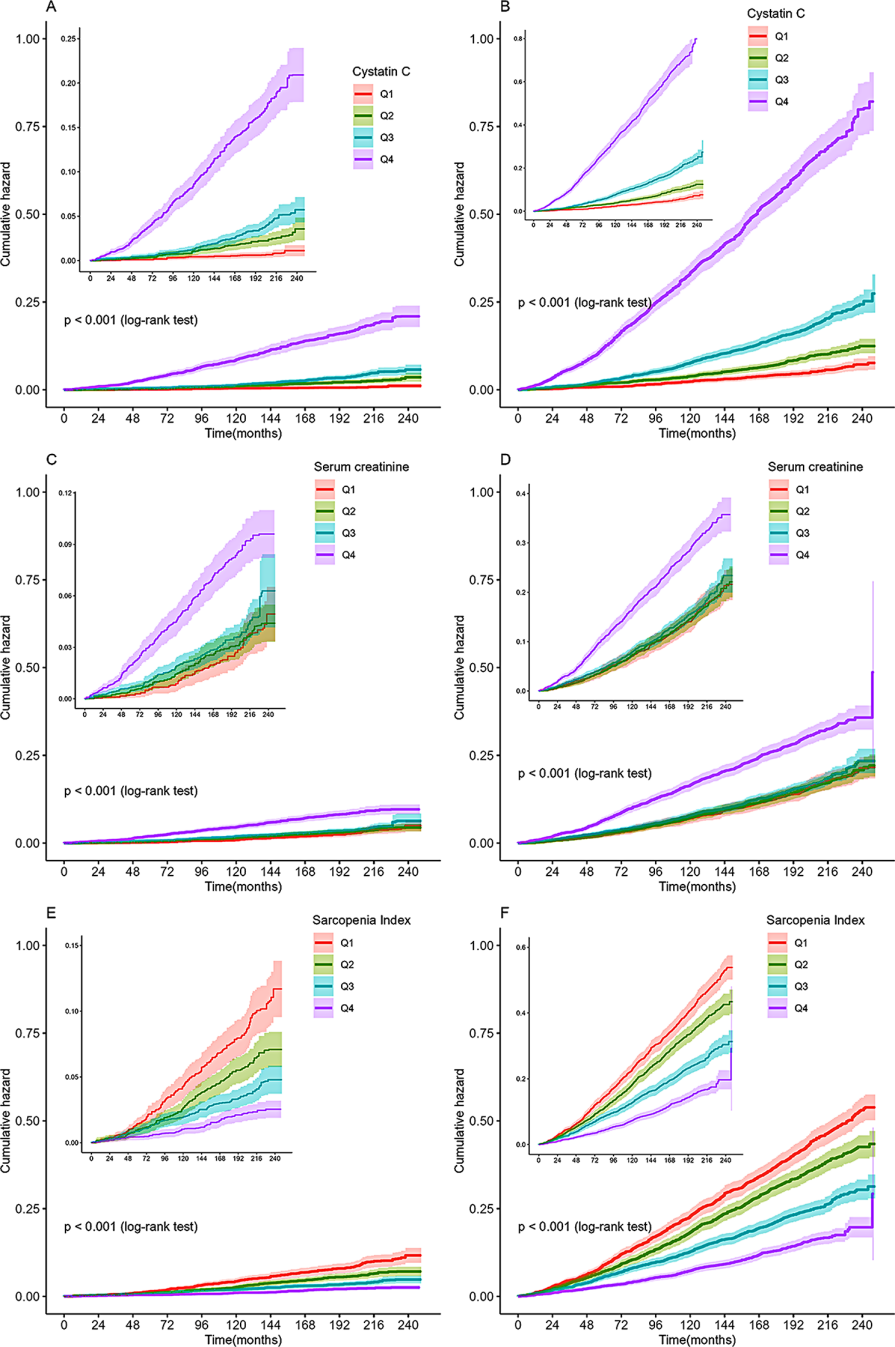
Cumulative death incidence curves. (**A**) cardiovascular death for cystatin C; (**B**) all-cause death for cystatin C; (**C**) cardiovascular death for serum creatinine; (**D**) all-cause death for serum creatinine; (**E**) cardiovascular death for Sarcopenia Index; (**F**) all-cause death for Sarcopenia Index

Kaplan‒Meier analysis revealed that participants with higher cystatin C and serum creatinine levels had a higher cumulative events incidence of cardiovascular and all-cause death (Fig. 2A and D). Meanwhile, elevated serum creatinine was still associated with higher cardiovascular and all-cause death by using unweighted data (Supplementary Fig. 1). Cox regression analysis was used to reveal the association of cystatin C and serum creatinine with cardiovascular and all-cause death. Elevated cystatin C levels were significantly associated with the increased risk of cardiovascular and all-cause death in all adjusted Models (Table 3). As compared with the first quartile in the fully adjusted Model, the highest cystatin C quartile had more than a twofold increased risk of cardiovascular death (HR: 3.36, 95% CI: 2.06–5.46, *P* < 0.001) and nearly a twofold increased risk of all-cause death (HR: 2.71, 95% CI: 2.17–3.38, *P* < 0.001). However, there wasn’t a significant association of serum creatinine with cardiovascular death and all-cause death in the fully adjusted Model (HR 0.89, 95% CI: 0.61–1.29; HR 0.83, 95% CI: 0.67–1.02, respectively). The significant association of serum creatinine with cardiovascular and all-cause death also weren’t observed by analyzing unweighted data (Supplementary Table 1).

**Table 3:** Results of Cox regression analysis

	Q1	Q2	Q3	Q4	*P* for trend
**Cystatin C**					
Cardiovascular death					
Model 1	Ref	2.12 (1.20–3.76)*	2.31 (1.41–3.77)***	5.29 (3.15–8.90)***	< 0.001
Model 2	Ref	2.13 (1.19–3.82)*	2.23 (1.36–3.65)**	4.63 (2.75–7.78)***	< 0.001
Model 3	Ref	2.17 (1.25–3.76)**	2.22 (1.40–3.53)***	3.36 (2.06–5.46)***	< 0.001
All-cause death					
Model 1	Ref	1.28 (1.02–1.60)*	1.72 (1.38–2.14)***	3.44 (2.75–4.31)***	< 0.001
Model 2	Ref	1.27 (1.01–1.61)*	1.64 (1.32–2.04)***	3.08 (2.46–3.86)***	< 0.001
Model 3	Ref	1.29 (1.02–1.63)**	1.67 (1.34–2.08)***	2.71 (2.17–3.38)***	< 0.001
**Serum creatinine**					
Cardiovascular death					
Model 1	Ref	0.92 (0.61–1.38)	0.97 (0.63–1.49)	1.60 (1.02–2.51)*	0.042
Model 2	Ref	0.96 (0.65–1.40)	0.99 (0.67–1.47)	1.59 (1.05–2.41)*	0.031
Model 3	Ref	0.90 (0.62–1.30)	0.80 (0.56–1.15)	0.89 (0.61–1.29)	0.431
All-cause death					
Model 1	Ref	0.89 (0.77–1.03)	0.83 (0.68–1.01)	1.16 (0.93–1.44)	0.311
Model 2	Ref	0.93 (0.81–1.06)	0.88 (0.73–1.05)	1.21 (0.99–1.48)	0.124
Model 3	Ref	0.87 (0.75–1.01)	0.74 (0.62–0.89)*	0.83 (0.67–1.02)	0.071
**Sarcopenia index**					
Cardiovascular death					
Model 1	Ref	0.68 (0.55–0.84)***	0.49 (0.38–0.64)***	0.37 (0.27–0.51)***	< 0.001
Model 2	Ref	0.71 (0.57–0.89)**	0.55 (0.42–0.71)***	0.41 (0.31–0.54)***	< 0.001
Model 3	Ref	0.66 (0.54–0.82)***	0.53 (0.40–0.69)***	0.41 (0.31–0.53)***	< 0.001
All-cause death					
Model 1	Ref	0.65 (0.58–0.73)***	0.47 (0.40–0.56)***	0.37 (0.30–0.45)***	< 0.001
Model 2	Ref	0.68 (0.61–0.76)***	0.52 (0.45–0.61)***	0.42 (0.35–0.50)***	< 0.001
Model 3	Ref	0.65 (0.59–0.73)***	0.50 (0.43–0.59)***	0.41 (0.35–0.49)***	< 0.001

Model 1: adjusted for age, sex

Model 2: Model 1 + adjusted for race/ethnicity, education, smoking status, drinking status, physical activity, and body mass index

Model 3: Model 2 + adjusted for total cholesterol, high density lipoprotein, albumin, alanine aminotransferase, blood urea nitrogen, blood uric acid, glucose, hypertension, diabetes mellites, and cardiovascular disease

*** *p* < 0.001, **p < 0.01, *p < 0.05. Values are presented as adjusted hazard ratio (95% confidence interval). The Q1-Q4 of cystatin C represent < 0.659, < 0.753, < 0.877 and ≥ 0.877; the Q1-Q4 of serum creatinine represent < 0.7, < 0.8, < 1.0 and ≥ 1.0; the Q1-Q4 of Sarcopenia Index represent < 88.41, < 106.24, < 125.52 and ≥ 125.52

**Fig. 3:**
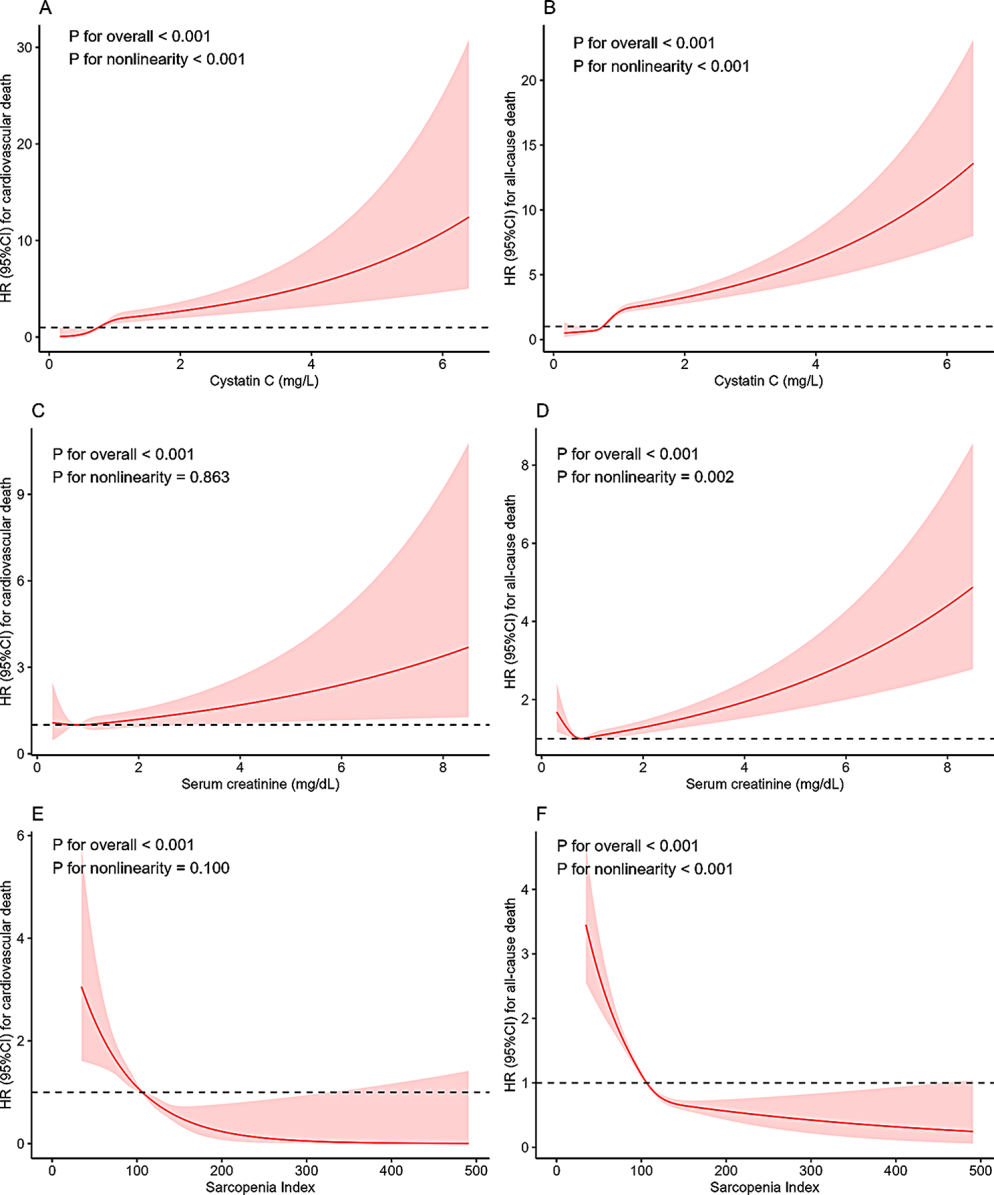
Restricted cubic splines. (**A**) cardiovascular death for cystatin C; (**B**) all-cause death for cystatin C; (**C**) cardiovascular death for serum creatinine; (**D**) all-cause death for serum creatinine; (**E**) cardiovascular death for Sarcopenia Index; (**F**) all-cause death for Sarcopenia Index

To explore the nonlinear association of cystatin C and serum creatinine with cardiovascular and all-cause death, the restricted cubic spline was performed (Fig. 3A and D). A nonlinear association of cystatin C with cardiovascular death and all-cause death was observed. The nonlinear association existed in all-cause death, while the linear association in cardiovascular death in serum creatinine. Additionally, serum creatinine seemed to have J-shaped relationship with the risk of all-cause death (Fig. 3D). The J-shaped relationship between serum creatinine and all-cause death persisted by analyzing unweighted data (Supplementary Fig. 2).

#### Sarcopenia index

The Kaplan‒Meier curves showed that compared with the fourth quartile of SI, participants with lower quartiles of SI had a higher cumulative risk of cardiovascular death and all-cause death (Fig. 2E and F). A significantly negative association of SI with cardiovascular and all-cause death was observed in Cox regression models (Table 3). After final adjustment for age, sex, race, education, smoking, drinking, physical activity, body mass index, total cholesterol, high density lipoprotein, albumin, alanine aminotransferase, blood urea nitrogen, blood uric acid, glucose, hypertension, diabetes mellites, and CVD (Model 3), compared to those with the first quartile of SI (< 88.41 vs. ≥125.52), participants with the highest quartile of SI were associated with reduced risk of cardiovascular death (HR: 0.41, 95% CI: 0.31–0.53, *P* < 0.001) and all-cause death (HR: 0.41, 95% CI: 0.35–0.49, *P* < 0.001). Moreover, there was a linear trend between SI and cardiovascular death (P for nonlinearity = 0.100), while a nonlinear association between SI and all-cause death (P for nonlinearity < 0.001) (Fig. 3E-F).

#### Subgroup analysis

**Fig. 4:**
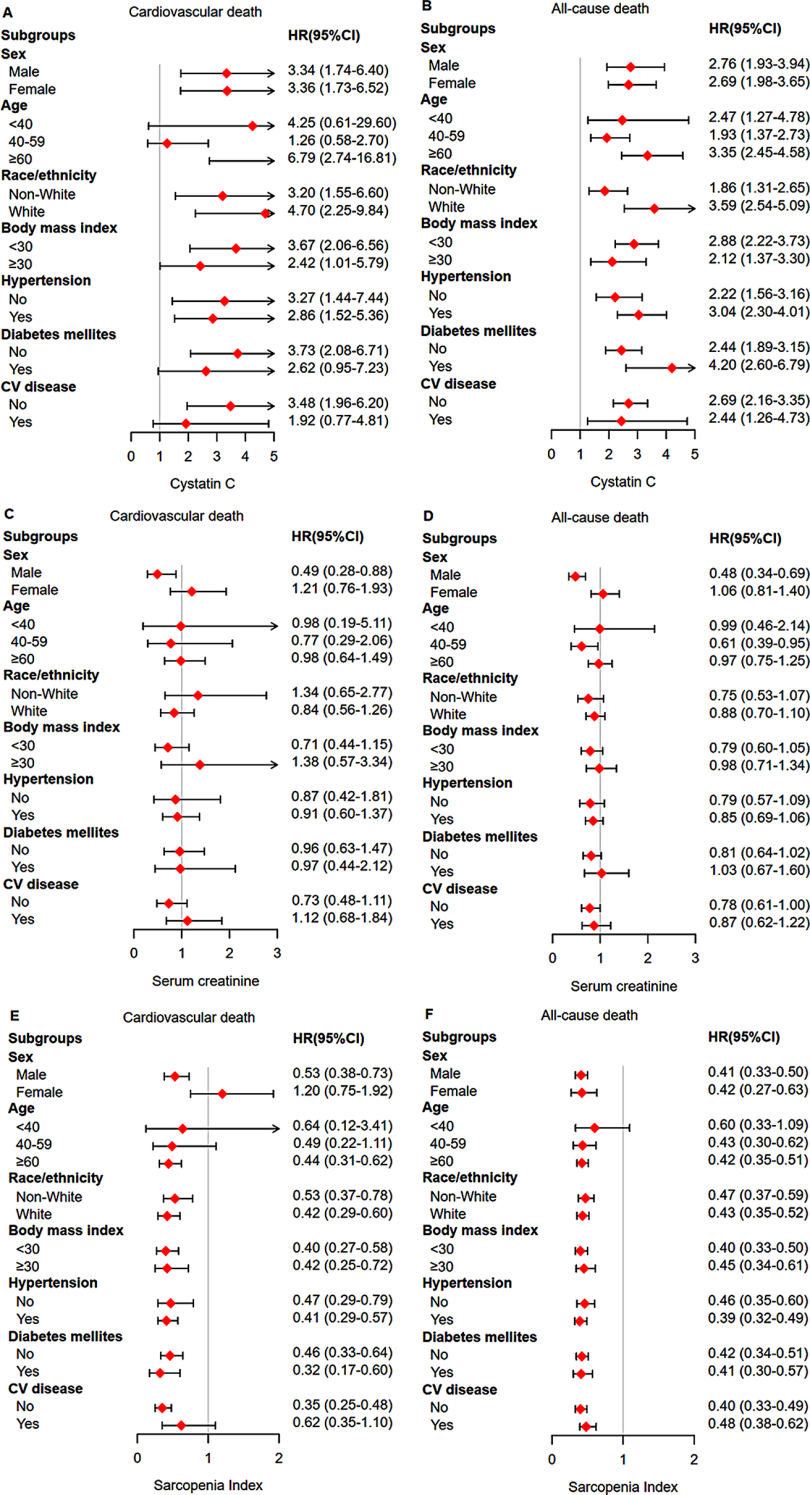
Cox regression subgroup analysis of the highest quartiles compared to the lowest quartiles. (**A**) cardiovascular death for cystatin C; (**B**) all-cause death for cystatin C; (**C**) cardiovascular death for serum creatinine; (**D**) all-cause death for serum creatinine; (**E**) cardiovascular death for Sarcopenia Index; (**F**) all-cause death for Sarcopenia Index. CV, cardiovascular

The results of subgroup analysis showed that the association of cystatin C, serum creatinine and SI with cardiovascular and all-cause death varied among different populations (Supplementary Tables 2–9). Figure 4 showed the results of comparing the highest and the lowest quartile of cystatin C, serum creatinine and SI.

For cardiovascular death, there was a significant interaction between cystatin C and race (P for interaction = 0.049). The adjusted HR of cardiovascular death (4.70 vs. 3.20) in the highest versus lowest cystatin C quartile in White participants was higher than in non-White participants, suggesting that Whites may be more susceptible than non-Whites to the effect of cystatin C on cardiovascular death. Additionally, there also were significant interaction of SI with race (P for interaction = 0.045), body mass index (P for interaction = 0.043) and CVD (P for interaction = 0.008). These findings may suggest that non-White individuals, those with obesity, and participants without CVD were more susceptible to cardiovascular death. There was also an interaction between serum creatinine and sex (P for interaction < 0.001); however, this interaction was not significant after analyzing unweighted data (P for interaction = 0.172). Furthermore, the association between cystatin C and cardiovascular death may be stronger in participants aged 60 years or older, those without diabetes mellitus, and those without CVD. A stronger association of SI with cardiovascular death was found in male and participants aged ≥ 60 years old.

For all-cause death, a significant interaction between cystatin C and race persisted (P for interaction = 0.006), indicating that Whites may still have a higher risk for all-cause death. There was only a significant interaction between SI and body mass index (P for interaction = 0.010). The adjusted HR of the highest vs. lowest SI quartile in obese participants was 0.45 (95% CI: 0.34–0.61), while that of the non-obese participants was 0.40 (95% CI: 0.33–0.50). This suggested that obese individuals may be more susceptible to the effect of SI on cardiovascular death than non-obese individuals. In addition, there was a significant interaction between serum creatinine and sex, whether using weighted (P for interaction = 0.002) or unweighted (P for interaction = 0.010) data analysis.

## Discussion

### Main findings

The main findings of this study are as follows: (1) There is a significant association of SI with the reduced risk of cardiovascular and all-cause death in a national representative cohort of US adults. (2) Elevated cystatin C is significantly associated with the higher risk of cardiovascular and all-cause death. (3) There is a nonlinear relationship between cystatin C and cardiovascular death as well as all-cause death, and a nonlinear relationship between SI and all-cause death.

### The relationship between cystatin C and serum creatinine and death

Previous research has investigated the correlation between cystatin C and death. It has been observed that cystatin C maintains an independent association with death in individuals with acute heart failure [11]. Additionally, preoperative levels of cystatin C could serve as robust predictors for death in patients undergoing coronary artery bypass grafting [12]. Furthermore, a meta-analysis also discovered that elevated levels of cystatin C were linked to a heightened risk of all cause and cardiovascular death among elderly individuals [10]. Wu et al. [20] also discovered that cystatin C demonstrates prognostic value for long-term death in patients aged over 40 years with relatively normal renal function, and a linear correlation between cystatin C and death outcomes was observed. However, our study using a restricted cubic spline revealed a nonlinear association between cystatin C levels and cardiovascular and all-cause death. And, the association between cystatin C and cardiovascular death was not observed in participants with age < 60 years by further age stratification analysis. Furthermore, cystatin C was a more precise indicator of death than serum creatinine, which identified cystatin C as a stronger predictor of cardiovascular and all-cause death compared to Serum creatinine among the elderly population [21]. Among patients without a history of CVD, cystatin C demonstrated superior predictive value for cardiovascular death compared to the estimated Glomerular Filtration Rate (eGFR) based on serum creatinine [22].

The specific mechanism underlying the link between cystatin C and long-term death remains unclear. This relationship could potentially be elucidated through the following: cystatin C, a lysosomal proteinase inhibitor, functions as an extracellular inhibitor of cysteine proteases. It has been suggested that cystatin C may be involved in the regulation of elastolytic cysteine proteases, such as cathepsins S and K, which could potentially contribute to the development of atheromatous plaques and subsequently increase the risk of cardiovascular events [23–25]. Previous studies have also shown that renal insufficiency was associated with an elevated risk of cardiovascular events and death [26, 27]. In terms of diagnostic accuracy and prognostic value, cystatin C has been found to outperform serum creatinine in identifying the early stages of chronic kidney disease and predicting unfavourable outcomes, such as renal failure and death [28]. Hence, individuals presenting with heightened levels of cystatin C may manifest early signs of renal insufficiency, thereby increasing their susceptibility to cardiovascular events.

### The association between sarcopenia index and death

Limited research has been conducted on the correlation between sarcopenia index (SI) and cardiovascular death. It has been found that lower SI is associated with an elevated risk of cardiovascular death in older patients who have undergone percutaneous coronary intervention [15]. Previous researches have primarily focused on examining the correlation between SI and death across various populations, including older individuals [14, 29], those with heart failure [16], and cancer patients [17, 18, 30]. Our study findings align with previous research in demonstrating a consistent association between SI and all-cause death.

Cystatin C and serum creatinine are readily obtainable and commonly employed indicators for evaluating renal function. However, serum creatinine concentration is significantly impacted by physiological and clinical factors that affect muscle mass [31]. Consequently, patients with reduced muscle mass or sarcopenia may exhibit lower levels of serum creatinine. In contrast, cystatin C is synthesized by all nucleated cells and remains unaffected by muscle metabolism [4]. These findings indicate that the Cre/CysC ratio may serve as a reliable surrogate measure for muscle mass. Previous research has similarly established that Cre/CysC can be regarded as a surrogate marker of muscle mass and is linked to death rates in the US population [9]. Consequently, we assume that the SI is a dependable and straightforward muscle marker for predicting death.

Our study has a main strength. this may be the first investigation to examine the correlation between SI and cardiovascular death in the American general population. Nevertheless, it is important to acknowledge several limitations in our study. Notably, the data relied on self-reported information, which may have introduced biases associated with self-selection and recall. A further limitation of our study design was that it may also be because cystatin C and serum creatinine were only measured once. Thus, we had no opportunity to assess changes in cystatin C or serum creatinine over time.

## Conclusions

In the general population in the United States, higher SI has been found to be significantly associated with the reduced long-term risk of cardiovascular and all-cause death. Meanwhile, there is also a significant positive association of cystatin C with cardiovascular and all-cause death. These findings may imply the potential important utility of cystatin C and SI in the cardiovascular risk estimation in general population in the United States, while the possibility of their application in other population still needs further prospective cohort studies to confirm.

### Electronic supplementary material

Below is the link to the electronic supplementary material.

Supplementary Material 1

Supplementary Material 2

## Data Availability

All data analysed in this study are publicly available at the National Centre for Health Statistics: https://www.cdc.gov/nchs/nhanes/.

## References

[1] Mensah GA, Roth GA, Fuster V. The Global Burden of Cardiovascular diseases and Risk factors: 2020 and Beyond. J Am Coll Cardiol. 2019;74(20):2529–32. 31727292 10.1016/j.jacc.2019.10.009

[2] Wang Z, Du A, Liu H, Wang Z, Hu J. Systematic analysis of the Global, Regional and National Burden of Cardiovascular diseases from 1990 to 2017. J Epidemiol Glob Health. 2022;12(1):92–103. 34902116 10.1007/s44197-021-00024-2 PMC8907368

[3] Roth GA, Mensah GA, Johnson CO, Addolorato G, Ammirati E, Baddour LM, Barengo NC, Beaton AZ, Benjamin EJ, Benziger CP, et al. Global Burden of Cardiovascular diseases and Risk factors, 1990–2019: Update from the GBD 2019 study. J Am Coll Cardiol. 2020;76(25):2982–3021. 33309175 10.1016/j.jacc.2020.11.010 PMC7755038

[4] Inker LA, Titan S. Measurement and estimation of GFR for Use in Clinical Practice: Core Curriculum 2021. Am J Kidney Dis. 2021;78(5):736–49. 34518032 10.1053/j.ajkd.2021.04.016

[5] Chew JS, Saleem M, Florkowski CM, George PM. Cystatin C–a paradigm of evidence based laboratory medicine. Clin Biochem Rev. 2008;29(2):47–62. 18787643 PMC2533150

[6] Park J, Mehrotra R, Rhee CM, Molnar MZ, Lukowsky LR, Patel SS, Nissenson AR, Kopple JD, Kovesdy CP, Kalantar-Zadeh K. Serum creatinine level, a surrogate of muscle mass, predicts mortality in peritoneal dialysis patients. Nephrol Dial Transpl. 2013;28(8):2146–55. 10.1093/ndt/gft213 PMC3765023 23743018

[7] Cruz-Jentoft AJ, Bahat G, Bauer J, Boirie Y, Bruyere O, Cederholm T, Cooper C, Landi F, Rolland Y, Sayer AA, et al. Sarcopenia: revised European consensus on definition and diagnosis. Age Ageing. 2019;48(1):16–31. 30312372 10.1093/ageing/afy169 PMC6322506

[8] Kashani KB, Frazee EN, Kukralova L, Sarvottam K, Herasevich V, Young PM, Kashyap R, Lieske JC. Evaluating muscle Mass by using markers of kidney function: development of the Sarcopenia Index. Crit Care Med. 2017;45(1):e23–9. 27611976 10.1097/CCM.0000000000002013

[9] Shi S, Jiang Y, Chen W, Chen K, Liao Y, Huang K. Diagnostic and prognostic value of the Creatinine/Cystatin C ratio for low muscle mass evaluation among US adults. Front Nutr. 2022;9:897774. 36017221 10.3389/fnut.2022.897774 PMC9398338

[10] Jung E, Ro YS, Ryu HH, Kong SY, Shin SD, Hwang SO. Cystatin C and mortality risk in the general population: systematic review and dose response meta-analysis. Biomarkers. 2022;27(3):222–9. 34847805 10.1080/1354750X.2021.1989489

[11] Breidthardt T, Sabti Z, Ziller R, Rassouli F, Twerenbold R, Kozhuharov N, Gayat E, Shrestha S, Barata S, Badertscher P, et al. Diagnostic and prognostic value of cystatin C in acute heart failure. Clin Biochem. 2017;50(18):1007–13. 28756070 10.1016/j.clinbiochem.2017.07.016

[12] Dardashti A, Nozohoor S, Algotsson L, Ederoth P, Bjursten H. The predictive value of s-cystatin C for mortality after coronary artery bypass surgery. J Thorac Cardiovasc Surg. 2016;152(1):139–46. 27056756 10.1016/j.jtcvs.2016.02.070

[13] Sun Y, Lu Q, Cheng B, Tao X. Prognostic value of cystatin C in patients with acute coronary syndrome: a systematic review and meta-analysis. Eur J Clin Invest. 2021;51(3):e13440. 33128232 10.1111/eci.13440

[14] Zhang L, Jin J, Tu YY, Zhao Z, Tao J, Zhang XY. Serum creatinine/cystatin C ratio is a predictor of all-cause mortality for older adults over 80 years. Heliyon. 2023;9(3):e14214. 36994407 10.1016/j.heliyon.2023.e14214 PMC10040501

[15] Lee HS, Park KW, Kang J, Ki YJ, Chang M, Han JK, Yang HM, Kang HJ, Koo BK, Kim HS. Sarcopenia Index as a predictor of clinical outcomes in older patients with coronary artery disease. J Clin Med 2020, 9(10). 10.3390/jcm9103121 PMC7600792 32992530

[16] Sunayama T, Fujimoto Y, Matsue Y, Dotare T, Daichi M, Yatsu S, Ishiwata S, Nakamura Y, Akama Y, Tsujimura Y, et al. Prognostic value of estimating appendicular muscle mass in heart failure using creatinine/cystatin C. Nutr Metab Cardiovasc Dis. 2023;33(9):1733–9. 37407312 10.1016/j.numecd.2023.05.031

[17] Ashton E, Arrondeau J, Jouinot A, Boudou-Rouquette P, Hirsch L, Huillard O, Ulmann G, Lupo-Mansuet A, Damotte D, Wislez M, et al. Impact of Sarcopenia indexes on survival and severe immune acute toxicity in metastatic non-small cell lung cancer patients treated with PD-1 immune checkpoint inhibitors. Clin Nutr. 2023;42(6):944–53. 37099986 10.1016/j.clnu.2023.03.023

[18] Jung CY, Kim HW, Han SH, Yoo TH, Kang SW, Park JT. Creatinine-cystatin C ratio and mortality in cancer patients: a retrospective cohort study. J Cachexia Sarcopenia Muscle. 2022;13(4):2064–72. 35478277 10.1002/jcsm.13006 PMC9397493

[19] Oftedal S, Aguiar EJ, Duncan MJ. Associations between multiple positive health behaviors and cardiometabolic risk using 3 alternative measures of physical activity: NHANES 2005–2006. Appl Physiol Nutr Metab. 2021;46(6):617–25. 33301364 10.1139/apnm-2020-0588

[20] Wu CK, Lin JW, Caffrey JL, Chang MH, Hwang JJ, Lin YS. Cystatin C and long-term mortality among subjects with normal creatinine-based estimated glomerular filtration rates: NHANES III (Third National Health and Nutrition Examination Survey). J Am Coll Cardiol. 2010;56(23):1930–6. 21109116 10.1016/j.jacc.2010.04.069

[21] Shlipak MG, Sarnak MJ, Katz R, Fried LF, Seliger SL, Newman AB, Siscovick DS, Stehman-Breen C. Cystatin C and the risk of death and cardiovascular events among elderly persons. N Engl J Med. 2005;352(20):2049–60. 15901858 10.1056/NEJMoa043161

[22] Svensson-Farbom P, Ohlson Andersson M, Almgren P, Hedblad B, Engstrom G, Persson M, Christensson A, Melander O. Cystatin C identifies cardiovascular risk better than creatinine-based estimates of glomerular filtration in middle-aged individuals without a history of cardiovascular disease. J Intern Med. 2014;275(5):506–21. 24279862 10.1111/joim.12169

[23] Taglieri N, Koenig W, Kaski JC. Cystatin C and cardiovascular risk. Clin Chem. 2009;55(11):1932–43. 19713275 10.1373/clinchem.2009.128397

[24] Liu J, Sukhova GK, Sun JS, Xu WH, Libby P, Shi GP. Lysosomal cysteine proteases in atherosclerosis. Arterioscler Thromb Vasc Biol. 2004;24(8):1359–66. 15178558 10.1161/01.ATV.0000134530.27208.41

[25] Sukhova GK, Shi GP, Simon DI, Chapman HA, Libby P. Expression of the elastolytic cathepsins S and K in human atheroma and regulation of their production in smooth muscle cells. J Clin Invest. 1998;102(3):576–83. 9691094 10.1172/JCI181 PMC508918

[26] Deferrari G, Cipriani A, La Porta E. Renal dysfunction in cardiovascular diseases and its consequences. J Nephrol. 2021;34(1):137–53. 32870495 10.1007/s40620-020-00842-w PMC7881972

[27] Huang G, Xu JB, Zhang TJ, Nie XL, Li Q, Liu Y, Lv Y, Wen RL, Yang L, Zhao BY. Hyperuricemia is associated with cardiovascular diseases clustering among very elderly women - a community based study in Chengdu, China. Sci Rep. 2017;7(1):996. 28428538 10.1038/s41598-017-01042-6 PMC5430531

[28] Spencer S, Desborough R, Bhandari S. Should Cystatin C eGFR become routine clinical practice? Biomolecules 2023, 13(7). 10.3390/biom13071075 PMC10377068 37509111

[29] Ren C, Su H, Tao J, Xie Y, Zhang X, Guo Q. Sarcopenia Index based on serum creatinine and cystatin C is Associated with Mortality, Nutritional Risk/Malnutrition and Sarcopenia in older patients. Clin Interv Aging. 2022;17:211–21. 35256845 10.2147/CIA.S351068 PMC8898017

[30] Zheng C, Wang E, Li JS, Xie K, Luo C, Ge QY, Hu LW, Shen Y. Serum creatinine/cystatin C ratio as a screening tool for Sarcopenia and prognostic indicator for patients with esophageal cancer. BMC Geriatr. 2022;22(1):207. 35287579 10.1186/s12877-022-02925-8 PMC8922862

[31] Stevens LA, Schmid CH, Greene T, Li L, Beck GJ, Joffe MM, Froissart M, Kusek JW, Zhang YL, Coresh J, et al. Factors other than glomerular filtration rate affect serum cystatin C levels. Kidney Int. 2009;75(6):652–60. 19119287 10.1038/ki.2008.638 PMC4557800

