# Supplementary material for: Cystatin C and sarcopenia index are associated with cardiovascular and all-cause death among adults in the United States
Source: BMC Public Health. 2024 Jul 23;24:1972. doi: 10.1186/s12889-024-19137-x (PMC11267836; doi:10.1186/s12889-024-19137-x)
Supplement: Supplementary file 1 — Supplementary Material 1 [file 12889_2024_19137_MOESM1_ESM.docx]

**Supplementary tables**

Supplementary Table 1 Unweighted results of Cox regression analysis for serum creatinine

|  | **Q1** | **Q2** | **Q3** | **Q4** | **P for trend** |
| --- | --- | --- | --- | --- | --- |
| **Cardiovascular death** |  |  |  |  |  |
| Model 1 | Ref | 1.23 (0.91-1.65) | 1.35 (1.01-1.79)* | 2.28 (1.73-3.02)*** | <0.001 |
| Model 2 | Ref | 1.21 (0.90-1.64) | 1.27 (0.95-1.69) | 1.99 (1.49-2.66)*** | <0.001 |
| Model 3 | Ref | 1.10 (0.82-1.49) | 0.98 (0.73-1.32) | 1.11 (0.82-1.51) | 0.677 |
| **All-cause death** |  |  |  |  |  |
| Model 1 | Ref | 1.08 (0.94-1.23) | 1.06 (0.93-1.21) | 1.59 (1.39-1.81)*** | <0.001 |
| Model 2 | Ref | 1.06 (0.93-1.22) | 1.02 (0.89-1.17) | 1.46 (1.27-1.68)*** | <0.001 |
| Model 3 | Ref | 0.98 (0.86-1.13) | 0.85 (0.74-0.98)* | 0.99 (0.86-1.15) | 0.873 |

Model 1: adjusted for age, sex.

Model 2: Model 1 + adjusted for race/ethnicity, education, smoking status, drinking status, physical activity, body mass index.

Model 3: Model 2 + adjusted for total cholesterol, high density lipoprotein, albumin, alanine aminotransferase, blood urea nitrogen, blood uric acid, glucose, hypertension, diabetes mellites, cardiovascular disease.

*** p<0.001, **p<0.01, *p<0.05. Values are presented as adjusted hazard ratio (95% confidence interval). The Q1-Q4 of serum creatinine represent <0.7, <0.8, <1.0 and ≥1.0

Supplementary Table 2 Results of cardiovascular death in subgroup analysis stratified by cystatin C quartile

| **Characteristics** | **Cystatin C** | | | | **P for trend** | **P for interaction** |
| --- | --- | --- | --- | --- | --- | --- |
|  | **Q1** | **Q2** | **Q3** | **Q4** |  |  |
|  |  | **HR（95%CI）** | | |  |  |
| **Sex** |  |  |  |  |  | 0.811 |
| Male (n=5003) | Ref | 2.08 (0.99-4.38) | 2.40 (1.41-4.09)** | 3.34 (1.74-6.40)*** | <0.001 |  |
| Female (n=4891) | Ref | 2.14 (0.92-4.97) | 1.94 (0.92-4.10) | 3.36 (1.73-6.52)*** | <0.001 |  |
| **Age** |  |  |  |  |  | 0.147 |
| <40 (n=3554) | Ref | 3.14 (0.59-16.83) | 4.36 (0.75-25.41) | 4.25 (0.61-29.60) | 0.136 |  |
| 40-59 (n=2956) | Ref | 1.03 (0.49-2.16) | 1.04 (0.53-2.01) | 1.26 (0.58-2.70) | 0.557 |  |
| ≥60 (n=3384) | Ref | 4.39 (1.41-13.65)* | 4.39 (1.74-11.04)** | 6.79 (2.74-16.81)*** | <0.001 |  |
| **Race/ethnicity** |  |  |  |  |  | 0.049 |
| No-White (n=4655) | Ref | 1.73 (0.78-3.80) | 1.27 (0.67-2.41) | 3.20 (1.55-6.60)** | 0.004 |  |
| White (n=5239) | Ref | 3.11 (1.39-6.98)** | 3.47 (1.70-7.06)*** | 4.70 (2.25-9.84)*** | <0.001 |  |
| **Body mass index** |  |  |  |  |  | 0.879 |
| <30 (n=6788) | Ref | 2.44 (1.18-5.02)* | 2.50 (1.40-4.45)** | 3.67 (2.06-6.56)*** | <0.001 |  |
| ≥30 (n=3106) | Ref | 1.43 (0.52-3.95) | 1.50 (0.72-3.11) | 2.42 (1.01-5.79)* | 0.032 |  |
| **Hypertension** |  |  |  |  |  | 0.478 |
| No (n=6390) | Ref | 2.66 (1.11-6.40)* | 2.17 (0.93-5.05) | 3.27 (1.44-7.44)* | 0.011 |  |
| Yes (n=3504) | Ref | 1.67 (0.86-3.24) | 1.94 (1.04-3.61)* | 2.86 (1.52-5.36)** | <0.001 |  |
| **Diabetes mellites** |  |  |  |  |  | 0.075 |
| No (n=8756) | Ref | 2.36 (1.17-4.73)* | 2.34 (1.26-4.33)** | 3.73 (2.08-6.71)*** | <0.001 |  |
| Yes (n=1138) | Ref | 1.82 (0.64-5.16) | 2.22 (0.99-4.96) | 2.62 (0.95-7.23) | 0.042 |  |
| **CV disease** |  |  |  |  |  | 0.087 |
| No (n=8881) | Ref | 2.69 (1.47-4.94)** | 2.12 (1.19-3.76)* | 3.48 (1.96-6.20)*** | <0.001 |  |
| Yes (n=1013) | Ref | 0.91 (0.27-3.00) | 1.64 (0.67-4.04) | 1.92 (0.77-4.81) | 0.056 |  |

Adjusted for age, sex, race/ethnicity, education, smoking status, drinking status, physical activity, body mass index, total cholesterol, high density lipoprotein, albumin, alanine aminotransferase, blood urea nitrogen, blood uric acid, glucose, hypertension, diabetes mellites, cardiovascular disease.

*** p<0.001, **p<0.01, *p<0.05. CV, cardiovascular.

Supplementary Table 3 Results of all-cause death in subgroup analysis stratified by cystatin C quartile

| **Characteristics** | **Cystatin C** | | | | **P for trend** | **P for interaction** |
| --- | --- | --- | --- | --- | --- | --- |
|  | **Q1** | **Q2** | **Q3** | **Q4** |  |  |
|  |  | **HR（95%CI）** | | |  |  |
| **Sex** |  |  |  |  |  | 0.500 |
| Male (n=5003) | Ref | 1.41 (0.99-2.02) | 1.67 (1.20-2.33)** | 2.76 (1.93-3.94)*** | <0.001 |  |
| Female (n=4891) | Ref | 1.17 (0.84-1.64) | 1.65 (1.22-2.25)** | 2.69 (1.98-3.65)*** | <0.001 |  |
| **Age** |  |  |  |  |  | 0.733 |
| <40 (n=3554) | Ref | 1.05 (0.58-1.89) | 1.14 (0.55-2.37) | 2.47 (1.27-4.78)** | 0.006 |  |
| 40-59 (n=2956) | Ref | 1.08 (0.74-1.59) | 1.34 (0.94-1.93) | 1.93 (1.37-2.73)*** | <0.001 |  |
| ≥60 (n=3384) | Ref | 1.56 (1.04-2.35)* | 2.05 (1.51-2.79)*** | 3.35 (2.45-4.58)*** | <0.001 |  |
| **Race/ethnicity** |  |  |  |  |  | 0.006 |
| No-White(n=4655) | Ref | 1.01 (0.70-1.47) | 1.21 (0.87-1.70) | 1.86 (1.31-2.65)*** | <0.001 |  |
| White (n=5239) | Ref | 1.66 (1.16-2.38)** | 2.21 (1.57-3.12)*** | 3.59 (2.54-5.09)*** | <0.001 |  |
| **Body mass index** |  |  |  |  |  | 0.362 |
| <30 (n=6788) | Ref | 1.34 (1.03-1.73)* | 1.76 (1.39-2.23)*** | 2.88 (2.22-3.73)*** | <0.001 |  |
| ≥30 (n=3106) | Ref | 1.04 (0.63-1.69) | 1.25 (0.81-1.93) | 2.12 (1.37-3.30)*** | <0.001 |  |
| **Hypertension** |  |  |  |  |  | 0.210 |
| No (n=6390) | Ref | 1.09 (0.77-1.55) | 1.38 (0.98-1.93) | 2.22 (1.56-3.16)*** | <0.001 |  |
| Yes (n=3504) | Ref | 1.49 (1.09-2.05)* | 1.89 (1.46-2.46)*** | 3.04 (2.30-4.01)*** | <0.001 |  |
| **Diabetes mellites** |  |  |  |  |  | 0.073 |
| No (n=8756) | Ref | 1.17 (0.89-1.54) | 1.48 (1.16-1.89)** | 2.44 (1.89-3.15)*** | <0.001 |  |
| Yes (n=1138) | Ref | 2.01 (1.13-3.57)* | 2.94 (1.83-4.74)*** | 4.20 (2.60-6.79)*** | <0.001 |  |
| **CV disease** |  |  |  |  |  | 0.137 |
| No (n=8881) | Ref | 1.29 (1.03-1.62)* | 1.57 (1.28-1.92)*** | 2.69 (2.16-3.35)*** | <0.001 |  |
| Yes (n=1013) | Ref | 1.21 (0.57-2.59) | 1.79 (0.92-3.48) | 2.44 (1.26-4.73)** | 0.002 |  |

Adjusted for age, sex, race/ethnicity, education, smoking status, drinking status, physical activity, body mass index, total cholesterol, high density lipoprotein, albumin, alanine aminotransferase, blood urea nitrogen, blood uric acid, glucose, hypertension, diabetes mellites, cardiovascular disease.

*** p<0.001, **p<0.01, *p<0.05. CV, cardiovascular.

Supplementary Table 4 Results of cardiovascular death in subgroup analysis stratified by serum creatinine quartile

| **Characteristics** | **Creatinine** | | | | **P for trend** | **P for interaction** |
| --- | --- | --- | --- | --- | --- | --- |
|  | **Q1** | **Q2** | **Q3** | **Q4** |  |  |
|  |  |  | **HR（95%CI）** |  |  |  |
| **Sex** |  |  |  |  |  | <0.001 |
| Male (n=5003) | Ref | 0.42 (0.22-0.82)* | 0.51 (0.27-0.95)* | 0.49 (0.28-0.88)* | 0.047 |  |
| Female (n=4891) | Ref | 1.12 (0.76-1.63) | 0.75 (0.45-1.27) | 1.21 (0.76-1.93) | 0.835 |  |
| **Age** |  |  |  |  |  | 0.145 |
| <40 (n=3554) | Ref | 0.66 (0.15-2.89) | 1.86 (0.50-6.95) | 0.98 (0.19-5.11) | 0.761 |  |
| 40-59 (n=2956) | Ref | 0.60 (0.25-1.40) | 1.06 (0.50-2.24) | 0.77 (0.29-2.06) | 0.885 |  |
| ≥60 (n=3384) | Ref | 1.06 (0.73-1.54) | 0.73 (0.47-1.14) | 0.98 (0.64-1.49) | 0.538 |  |
| **Race/ethnicity** |  |  |  |  |  | 0.291 |
| No-White (n=4655) | Ref | 1.60 (0.84-3.03) | 0.99 (0.49-2.00) | 1.34 (0.65-2.77) | 0.740 |  |
| White (n=5239) | Ref | 0.78 (0.52-1.17) | 0.78 (0.52-1.17) | 0.84 (0.56-1.26) | 0.429 |  |
| **Body mass index** |  |  |  |  |  | 0.512 |
| <30 (n=6788) | Ref | 0.82 (0.54-1.25) | 0.70 (0.48-1.04) | 0.71 (0.44-1.15) | 0.123 |  |
| ≥30 (n=3106) | Ref | 1.07 (0.56-2.05) | 1.12 (0.59-2.13) | 1.38 (0.57-3.34) | 0.479 |  |
| **Hypertension** |  |  |  |  |  | 0.989 |
| No (n=6390) | Ref | 0.94 (0.53-1.67) | 0.86 (0.42-1.75) | 0.87 (0.42-1.81) | 0.662 |  |
| Yes (n=3504) | Ref | 0.86 (0.53-1.39) | 0.78 (0.50-1.22) | 0.91 (0.60-1.37) | 0.549 |  |
| **Diabetes mellites** |  |  |  |  |  | 0.531 |
| No (n=8756) | Ref | 0.97 (0.68-1.40) | 0.94 (0.61-1.43) | 0.96 (0.63-1.47) | 0.826 |  |
| Yes (n=1138) | Ref | 0.79 (0.37-1.67) | 0.62 (0.28-1.37) | 0.97 (0.44-2.12) | 0.785 |  |
| **CV disease** |  |  |  |  |  | 0.662 |
| No (n=8881) | Ref | 0.80 (0.53-1.20) | 0.76 (0.50-1.15) | 0.73 (0.48-1.11) | 0.138 |  |
| Yes (n=1013) | Ref | 1.15 (0.65-2.01) | 0.86 (0.49-1.49) | 1.12 (0.68-1.84) | 0.960 |  |

Adjusted for age, sex, race/ethnicity, education, smoking status, drinking status, physical activity, body mass index, total cholesterol, high density lipoprotein, albumin, alanine aminotransferase, blood urea nitrogen, blood uric acid, glucose, hypertension, diabetes mellites, cardiovascular disease.

*** p<0.001, **p<0.01, *p<0.05. CV, cardiovascular.

Supplementary Table 5 Results of all-cause death in subgroup analysis stratified by serum creatinine quartile

| **Characteristics** | **Creatinine** | | | | **P for trend** | **P for interaction** |
| --- | --- | --- | --- | --- | --- | --- |
|  | **Q1** | **Q2** | **Q3** | **Q4** |  |  |
|  |  |  | **HR（95%CI）** |  |  |  |
| **Sex** |  |  |  |  |  | 0.002 |
| Male (n=5003) | Ref | 0.60 (0.46-0.80)*** | 0.44 (0.31-0.63)*** | 0.48 (0.34-0.69)*** | <0.001 |  |
| Female (n=4891) | Ref | 0.88 (0.71-1.08) | 0.85 (0.67-1.07) | 1.06 (0.81-1.40) | 0.751 |  |
| **Age** |  |  |  |  |  | 0.743 |
| <40 (n=3554) | Ref | 0.73 (0.41-1.28) | 0.63 (0.36-1.12) | 0.99 (0.46-2.14) | 0.890 |  |
| 40-59 (n=2956) | Ref | 0.68 (0.49-0.95)* | 0.65 (0.47-0.90)** | 0.61 (0.39-0.95)* | 0.025 |  |
| ≥60 (n=3384) | Ref | 1.03 (0.84-1.27) | 0.85 (0.65-1.09) | 0.97 (0.75-1.25) | 0.501 |  |
| **Race/ethnicity** |  |  |  |  |  | 0.588 |
| No-White (n=4655) | Ref | 0.90 (0.64-1.27) | 0.72 (0.55-0.95) | 0.75 (0.53-1.07) | 0.043 |  |
| White (n=5239) | Ref | 0.89 (0.75-1.05) | 0.77 (0.62-0.96)* | 0.88 (0.70-1.10) | 0.153 |  |
| **Body mass index** |  |  |  |  |  | 0.982 |
| <30 (n=6788) | Ref | 0.87 (0.70-1.09) | 0.72 (0.57-0.92)** | 0.79 (0.60-1.05) | 0.054 |  |
| ≥30 (n=3106) | Ref | 0.94 (0.72-1.23) | 0.87 (0.68-1.11) | 0.98 (0.71-1.34) | 0.770 |  |
| **Hypertension** |  |  |  |  |  | 0.922 |
| No (n=6390) | Ref | 0.83 (0.65-1.07) | 0.74 (0.53-1.02) | 0.79 (0.57-1.09) | 0.121 |  |
| Yes (n=3504) | Ref | 0.89 (0.75-1.07) | 0.75 (0.62-0.90) | 0.85 (0.69-1.06) | 0.085 |  |
| **Diabetes mellites** |  |  |  |  |  | 0.895 |
| No (n=8756) | Ref | 0.88 (0.74-1.04) | 0.73 (0.57-0.92)** | 0.81 (0.64-1.02) | 0.036 |  |
| Yes (n=1138) | Ref | 0.81 (0.53-1.25) | 0.88 (0.58-1.34) | 1.03 (0.67-1.60) | 0.788 |  |
| **CV disease** |  |  |  |  |  | 0.240 |
| No (n=8881) | Ref | 0.86 (0.73-1.02) | 0.76 (0.62-0.93)** | 0.78 (0.61-1.00) | 0.035 |  |
| Yes (n=1013) | Ref | 0.94 (0.65-1.35) | 0.64 (0.46-0.90)** | 0.87 (0.62-1.22) | 0.151 |  |

Adjusted for age, sex, race/ethnicity, education, smoking status, drinking status, physical activity, body mass index, total cholesterol, high density lipoprotein, albumin, alanine aminotransferase, blood urea nitrogen, blood uric acid, glucose, hypertension, diabetes mellites, cardiovascular disease.

*** p<0.001, **p<0.01, *p<0.05. CV, cardiovascular.

Supplementary Table 6 Results of cardiovascular death in subgroup analysis stratified by sarcopenia index quartile

| **Characteristics** | **Sarcopenia Index** | | | | **P for trend** | **P for interaction** |
| --- | --- | --- | --- | --- | --- | --- |
|  | **Q1** | **Q2** | **Q3** | **Q4** |  |  |
|  |  |  | **HR（95%CI）** |  |  |  |
| **Sex** |  |  |  |  |  | 0.060 |
| Male (n=5003) | Ref | 0.96 (0.69-1.33) | 0.64 (0.43-0.94)* | 0.53 (0.38-0.73)*** | <0.001 |  |
| Female (n=4891) | Ref | 1.11 (0.76-1.63) | 0.75 (0.45-1.26) | 1.20 (0.75-1.92) | 0.001 |  |
| **Age** |  |  |  |  |  | 0.807 |
| <40 (n=3554) | Ref | 0.60 (0.09-3.87) | 1.29 (0.31-5.35) | 0.64 (0.12-3.41) | 0.846 |  |
| 40-59 (n=2956) | Ref | 0.75 (0.46-1.23) | 0.73 (0.37-1.43) | 0.49 (0.22-1.11) | 0.114 |  |
| ≥60 (n=3384) | Ref | 0.71 (0.54-0.92)** | 0.50 (0.34-0.72)*** | 0.44 (0.31-0.62)*** | <0.001 |  |
| **Race/ethnicity** |  |  |  |  |  | 0.045 |
| No-White (n=4655) | Ref | 0.66 (0.38-1.13) | 0.94 (0.60-1.46) | 0.53 (0.37-0.78)** | 0.018 |  |
| White (n=5239) | Ref | 0.70 (0.55-0.88)** | 0.48 (0.33-0.69)*** | 0.42 (0.29-0.60)*** | <0.001 |  |
| **Body mass index** |  |  |  |  |  | 0.043 |
| <30 (n=6788) | Ref | 0.65 (0.50-0.85)** | 0.43 (0.31-0.59)*** | 0.40 (0.27-0.58)*** | <0.001 |  |
| ≥30 (n=3106) | Ref | 0.69 (0.49-0.97)* | 0.81 (0.50-1.29) | 0.42 (0.25-0.72)*** | 0.007 |  |
| **Hypertension** |  |  |  |  |  | 0.254 |
| No (n=6390) | Ref | 0.89 (0.57-1.38) | 0.58 (0.36-0.91)* | 0.47 (0.29-0.79)** | <0.001 |  |
| Yes (n=3504) | Ref | 0.58 (0.45-0.75)*** | 0.52 (0.39-0.71)*** | 0.41 (0.29-0.57)*** | <0.001 |  |
| **Diabetes mellites** |  |  |  |  |  | 0.645 |
| No (n=8756) | Ref | 0.66 (0.51-0.85)** | 0.54 (0.37-0.78)** | 0.46 (0.33-0.64)*** | <0.001 |  |
| Yes (n=1138) | Ref | 0.66 (0.44-0.99)* | 0.61 (0.38-0.96)* | 0.32 (0.17-0.60)*** | <0.001 |  |
| **CV disease** |  |  |  |  |  | 0.008 |
| No (n=8881) | Ref | 0.65 (0.47-0.91)* | 0.39 (0.27-0.54)*** | 0.35 (0.25-0.48)*** | <0.001 |  |
| Yes (n=1013) | Ref | 0.74 (0.47-1.15) | 0.92 (0.62-1.37) | 0.62 (0.35-1.10) | 0.184 |  |

Adjusted for age, sex, race/ethnicity, education, smoking status, drinking status, physical activity, body mass index, total cholesterol, high density lipoprotein, albumin, alanine aminotransferase, blood urea nitrogen, blood uric acid, glucose, hypertension, diabetes mellites, cardiovascular disease.

*** p<0.001, **p<0.01, *p<0.05. CV, cardiovascular.

Supplementary Table 7 Results of all-cause death in subgroup analysis stratified by sarcopenia index quartile

| **Characteristics** | **Sarcopenia Index** | | | | **P for trend** | **P for interaction** |
| --- | --- | --- | --- | --- | --- | --- |
|  | **Q1** | **Q2** | **Q3** | **Q4** |  |  |
|  |  |  | **HR（95%CI）** |  |  |  |
| **Sex** |  |  |  |  |  | 0.954 |
| Male (n=5003) | Ref | 0.67 (0.57-0.79)*** | 0.50 (0.41-0.61)*** | 0.41 (0.33-0.50)*** | <0.001 |  |
| Female (n=4891) | Ref | 0.64 (0.55-0.74)*** | 0.51 (0.41-0.65)*** | 0.42 (0.27-0.63)*** | <0.001 |  |
| **Age** |  |  |  |  |  | 0.213 |
| <40 (n=3554) | Ref | 0.52 (0.28-0.95)* | 0.68 (0.37-1.28) | 0.60 (0.33-1.09) | 0.231 |  |
| 40-59 (n=2956) | Ref | 0.64 (0.49-0.85)** | 0.48 (0.37-0.63)*** | 0.43 (0.30-0.62)*** | <0.001 |  |
| ≥60 (n=3384) | Ref | 0.70 (0.62-0.80)*** | 0.52 (0.43-0.63)*** | 0.42 (0.35-0.51)*** | <0.001 |  |
| **Race/ethnicity** |  |  |  |  |  | 0.116 |
| No-White(n=4655) | Ref | 0.68 (0.52-0.89)** | 0.63 (0.49-0.82)*** | 0.47 (0.37-0.59)*** | <0.001 |  |
| White (n=5239) | Ref | 0.67 (0.58-0.77)*** | 0.49 (0.40-0.60)*** | 0.43 (0.35-0.52)*** | <0.001 |  |
| **Body mass index** |  |  |  |  |  | 0.010 |
| <30 (n=6788) | Ref | 0.66 (0.58-0.76)*** | 0.46 (0.39-0.54)*** | 0.40 (0.33-0.50)*** | <0.001 |  |
| ≥30 (n=3106) | Ref | 0.67 (0.54-0.84)*** | 0.66 (0.50-0.86)** | 0.45 (0.34-0.61)*** | <0.001 |  |
| **Hypertension** |  |  |  |  |  | 0.980 |
| No (n=6390) | Ref | 0.66 (0.53-0.82)*** | 0.54 (0.42-0.68)*** | 0.46 (0.35-0.60)*** | <0.001 |  |
| Yes (n=3504) | Ref | 0.65 (0.57-0.74)*** | 0.49 (0.41-0.59)*** | 0.39 (0.32-0.49)*** | <0.001 |  |
| **Diabetes mellites** |  |  |  |  |  | 0.493 |
| No (n=8756) | Ref | 0.65 (0.58-0.73)*** | 0.49 (0.41-0.59)*** | 0.42 (0.34-0.51)*** | <0.001 |  |
| Yes (n=1138) | Ref | 0.67 (0.54-0.84)*** | 0.59 (0.45-0.77)*** | 0.41 (0.30-0.57)*** | <0.001 |  |
| **CV disease** |  |  |  |  |  | 0.730 |
| No (n=8881) | Ref | 0.67 (0.59-0.76)*** | 0.49 (0.42-0.58)*** | 0.40 (0.33-0.49)*** | <0.001 |  |
| Yes (n=1013) | Ref | 0.63 (0.52-0.77)*** | 0.57 (0.43-0.75)*** | 0.48 (0.38-0.62)*** | <0.001 |  |

Adjusted for age, sex, race/ethnicity, education, smoking status, drinking status, physical activity, body mass index, total cholesterol, high density lipoprotein, albumin, alanine aminotransferase, blood urea nitrogen, blood uric acid, glucose, hypertension, diabetes mellites, cardiovascular disease.

*** p<0.001, **p<0.01, *p<0.05. CV, cardiovascular.

Supplementary Table 8 Unweighted results of cardiovascular death in subgroup analysis stratified by serum creatinine quartile

| **Characteristics** | **Creatinine** | | | | **P for trend** | **P for interaction** |
| --- | --- | --- | --- | --- | --- | --- |
|  | **Q1** | **Q2** | **Q3** | **Q4** |  |  |
|  |  |  | **HR（95%CI）** |  |  |  |
| **Sex** |  |  |  |  |  | 0.172 |
| Male (n=5003) | Ref | 0.97 (0.53-1.77) | 0.93 (0.52-1.65) | 0.95 (0.54-1.70) | 0.839 |  |
| Female (n=4891) | Ref | 1.13 (0.79-1.63) | 0.87 (0.60-1.28) | 1.32 (0.88-1.98) | 0.379 |  |
| **Age** |  |  |  |  |  | 0.735 |
| <40 (n=3554) | Ref | 0.58 (0.09-3.74) | 2.04 (0.41-10.17) | 1.50 (0.25-9.14) | 0.406 |  |
| 40-59 (n=2956) | Ref | 1.18 (0.54-2.57) | 1.10 (0.50-2.42) | 0.86 (0.36-2.07) | 0.712 |  |
| ≥60 (n=3384) | Ref | 1.11 (0.79-1.55) | 0.95 (0.69-1.32) | 1.15 (0.82-1.62) | 0.608 |  |
| **Race/ethnicity** |  |  |  |  |  | 0.298 |
| No-White (n=4655) | Ref | 1.57 (1.00-2.48) | 1.21 (0.75-1.93) | 1.42 (0.88-2.30) | 0.313 |  |
| White (n=5239) | Ref | 0.85 (0.57-1.27) | 0.86 (0.59-1.26) | 0.99 (0.67-1.46) | 0.969 |  |
| **Body mass index** |  |  |  |  |  | 0.225 |
| <30 (n=6788) | Ref | 0.91 (0.63-1.31) | 0.91 (0.64-1.30) | 0.95 (0.65-1.37) | 0.785 |  |
| ≥30 (n=3106) | Ref | 1.65 (0.96-2.84) | 1.21 (0.70-2.07) | 1.59 (0.91-2.76) | 0.231 |  |
| **Hypertension** |  |  |  |  |  | 0.692 |
| No (n=6390) | Ref | 0.94 (0.55-1.60) | 0.87 (0.51-1.47) | 0.97 (0.55-1.69) | 0.84 |  |
| Yes (n=3504) | Ref | 1.15 (0.80-1.66) | 1.00 (0.70-1.43) | 1.14 (0.79-1.65) | 0.67 |  |
| **Diabetes mellites** |  |  |  |  |  | 0.286 |
| No (n=8756) | Ref | 1.04 (0.73-1.46) | 1.04 (0.75-1.46) | 1.12 (0.78-1.59) | 0.551 |  |
| Yes (n=1138) | Ref | 1.40 (0.76-2.60) | 0.93 (0.50-1.73) | 1.30 (0.70-2.43) | 0.713 |  |
| **CV disease** |  |  |  |  |  | 0.677 |
| No (n=8881) | Ref | 1.05 (0.74-1.50) | 0.98 (0.69-1.39) | 1.04 (0.71-1.52) | 0.934 |  |
| Yes (n=1013) | Ref | 1.24 (0.69-2.20) | 0.93 (0.54-1.62) | 1.10 (0.63-1.90) | 0.995 |  |

Adjusted for age, sex, race/ethnicity, education, smoking status, drinking status, physical activity, body mass index, total cholesterol, high density lipoprotein, albumin, alanine aminotransferase, blood urea nitrogen, blood uric acid, glucose, hypertension, diabetes mellites, cardiovascular disease.

*** p<0.001, **p<0.01, *p<0.05. CV, cardiovascular.

Supplementary Table 9 Unweighted results of all-cause death in subgroup analysis stratified by serum creatinine quartile

| **Characteristics** | **Creatinine** | | | | **P for trend** | **P for interaction** |
| --- | --- | --- | --- | --- | --- | --- |
|  | **Q1** | **Q2** | **Q3** | **Q4** |  |  |
|  |  |  | **HR（95%CI）** |  |  |  |
| **Sex** |  |  |  |  |  | 0.010 |
| Male (n=5003) | Ref | 0.84 (0.65-1.09) | 0.65 (0.50-0.84)** | 0.73 (0.56-0.94)* | 0.003 |  |
| Female (n=4891) | Ref | 0.99 (0.83-1.18) | 0.94 (0.79-1.12) | 1.22 (1.00-1.50) | 0.091 |  |
| **Age** |  |  |  |  |  | 0.538 |
| <40 (n=3554) | Ref | 0.96 (0.54-1.70) | 0.80 (0.43-1.48) | 1.10 (0.55-2.23) | 0.923 |  |
| 40-59 (n=2956) | Ref | 0.79 (0.58-1.08) | 0.69 (0.50-0.95)* | 0.60 (0.42-0.86)** | 0.005 |  |
| ≥60 (n=3384) | Ref | 1.05 (0.89-1.23) | 0.92 (0.78-1.08) | 1.11 (0.94-1.31) | 0.510 |  |
| **Race/ethnicity** |  |  |  |  |  | 0.837 |
| No-White (n=4655) | Ref | 1.07 (0.88-1.30) | 0.94 (0.76-1.15) | 1.06 (0.85-1.32) | 0.902 |  |
| White (n=5239) | Ref | 0.95 (0.78-1.15) | 0.84 (0.69-1.01) | 1.00 (0.82-1.21) | 0.666 |  |
| **Body mass index** |  |  |  |  |  | 0.484 |
| <30 (n=6788) | Ref | 0.90 (0.76-1.07) | 0.80 (0.68-0.95)** | 0.90 (0.76-1.08) | 0.139 |  |
| ≥30 (n=3106) | Ref | 1.26 (0.98-1.62) | 1.04 (0.81-1.33) | 1.26 (0.97-1.64) | 0.247 |  |
| **Hypertension** |  |  |  |  |  | 0.243 |
| No (n=6390) | Ref | 0.80 (0.64-1.00) | 0.74 (0.59-0.93)* | 0.82 (0.64-1.05) | 0.085 |  |
| Yes (n=3504) | Ref | 1.12 (0.94-1.34) | 0.92 (0.77-1.10) | 1.09 (0.90-1.31) | 0.856 |  |
| **Diabetes mellites** |  |  |  |  |  | 0.969 |
| No (n=8756) | Ref | 0.96 (0.82-1.12) | 0.83 (0.71-0.98)* | 0.92 (0.78-1.09) | 0.156 |  |
| Yes (n=1138) | Ref | 1.06 (0.78-1.44) | 0.98 (0.73-1.33) | 1.31 (0.96-1.78) | 0.145 |  |
| **CV disease** |  |  |  |  |  | 0.863 |
| No (n=8881) | Ref | 0.98 (0.84-1.15) | 0.85 (0.73-0.99)* | 0.94 (0.80-1.12) | 0.237 |  |
| Yes (n=1013) | Ref | 1.04 (0.74-1.44) | 0.82 (0.60-1.13) | 1.03 (0.76-1.42) | 0.800 |  |

Adjusted for age, sex, race/ethnicity, education, smoking status, drinking status, physical activity, body mass index, total cholesterol, high density lipoprotein, albumin, alanine aminotransferase, blood urea nitrogen, blood uric acid, glucose, hypertension, diabetes mellites, cardiovascular disease.

*** p<0.001, **p<0.01, *p<0.05. CV, cardiovascular.
