# Supplementary material for: Cystatin C and sarcopenia index are associated with cardiovascular and all-cause death among adults in the United States
Source: BMC Public Health. 2024 Jul 23;24:1972. doi: 10.1186/s12889-024-19137-x (PMC11267836; doi:10.1186/s12889-024-19137-x)
Supplement: Supplementary file 2 — Supplementary Material 2 [file 12889_2024_19137_MOESM2_ESM.docx]

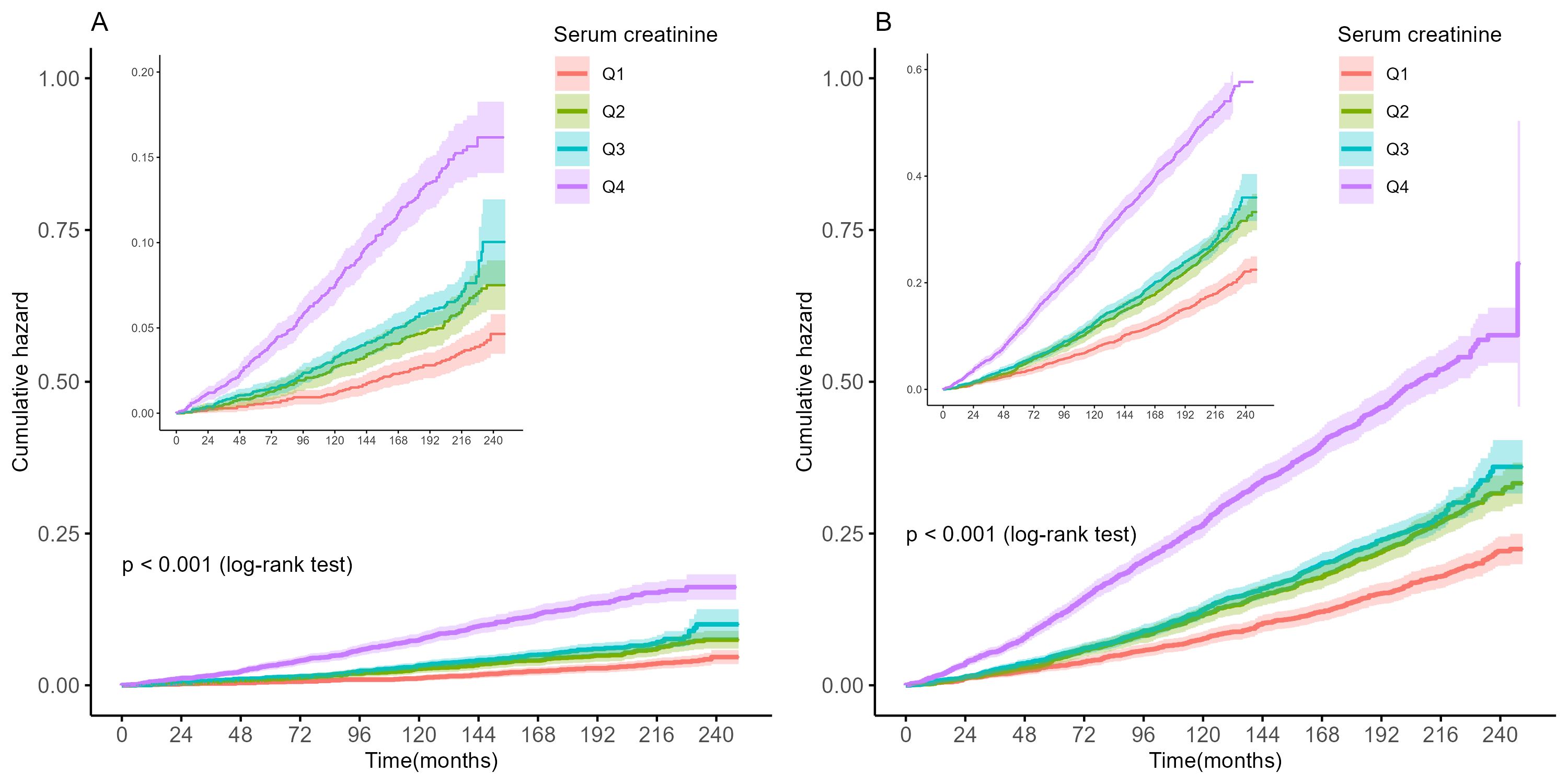


**Supplementary Fig.1 Cumulative death incidence curves according to unweighted data of serum creatinine. A cardiovascular death；B all-cause death**


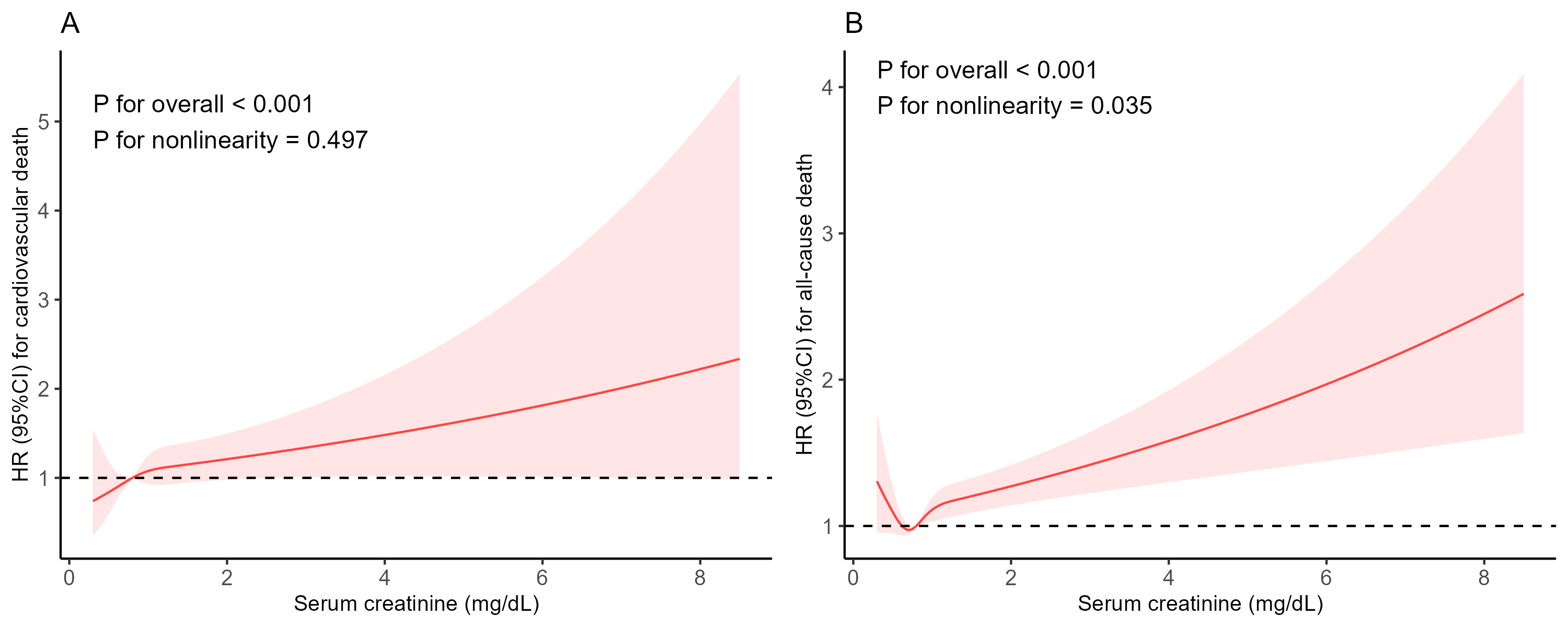


**Supplementary Fig.2** **Restricted cubic splines according to unweighted data of serum creatinine. A cardiovascular death；B all-cause death**
